# Comparison of the neural correlates of encoding item-item and item-context associations

**DOI:** 10.3389/fnhum.2013.00436

**Published:** 2013-08-14

**Authors:** Jenny X. Wong, Marianne de Chastelaine, Michael D. Rugg

**Affiliations:** Center for Vital Longevity and School of Behavioral and Brain Sciences, The University of Texas at DallasDallas, TX, USA

**Keywords:** associative memory, source memory, fMRI, subsequent memory paradigm, episodic encoding

## Abstract

fMRI was employed to investigate the role of the left inferior frontal gyrus (LIFG) in the encoding of item-item and item-context associations. On each of a series of study trials subjects viewed a picture that was presented either to the left or right of fixation, along with a subsequently presented word that appeared at fixation. Memory was tested in a subsequent memory test that took place outside of the scanner. On each test trial one of two forced choice judgments was required. For the associative test, subjects chose between the word paired with the picture at study and a word studied on a different trial. For the source test, the judgment was whether the picture had been presented on the left or right. Successful encoding of associative information was accompanied by subsequent memory effects in several cortical regions, including much of the LIFG. By contrast, successful source encoding was selectively associated with a subsequent memory effect in right fusiform cortex. The finding that the LIFG was enhanced during successful associative, but not source, encoding is interpreted in light of the proposal that subsequent memory effects are localized to cortical regions engaged by the on-line demands of the study task.

## Introduction

Episodic memory—memory for a unique event—depends upon the ability to encode associations between the different components that comprise the event (Tulving, 1983). In an experimental setting these components often include study items such as words or pictures that are presented in association with specific contextual information, such as the location on the display monitor where an item is presented. Associations can be formed between two or more items belonging to a study event (item-item associations, or “*associative*” memory), or between an item and one or more contextual features (item-context associations, or “*source*” memory). As is described below, fMRI has been employed to investigate the neural correlates of encoding both of these types of association.

fMRI studies of the neural correlates of successful episodic encoding have almost invariably utilized the “subsequent memory procedure” (Paller and Wagner, 2002), which permits identification of brain regions where study activity varies according to performance on a later memory test. Across a variety of different study materials and tasks, it has consistently been reported that successful encoding of item-item associations (as operationalized by accurate performance on a later associative recognition test) is associated with enhanced activity in the medial temporal lobe (MTL), including the hippocampus and, among other lateral prefrontal cortex regions, the middle and ventral aspects of the left inferior frontal gyrus (LIFG) (e.g., Sperling et al., 2003; Jackson and Schacter, 2004; Prince et al., 2005; Chua et al., 2007; Park and Rugg, 2008; Blumenfeld et al., 2011). Subsequent associative memory effects have also been reported in cortical regions other than the IFG. In one study (Blumenfeld et al., 2011), successful associative, rather than item, encoding was selectively indexed by subsequent memory effects in dorsolateral prefrontal cortex (i.e., the middle rather than the inferior frontal gyrus). This region has not, however, consistently been identified in studies of associative encoding (Kim, 2011). In two studies that investigated whether these effects varied according to study material [face-house pairs in Summerfield et al. (2006), and picture pairs vs. word pairs in Park and Rugg (2011)], subsequent memory effects were identified in regions that were preferentially engaged by the respective classes of study item (the “fusiform face” and “parahippocampal place” areas in Summerfield et al. (2006), for example). These findings are consistent with the proposal that cortical subsequent memory effects reflect modulation of activity in regions engaged during the on-line processing of a study event (Rugg et al., 2008).

A second strand of research has focused on identifying the neural correlates of the successful encoding of source memories (e.g., Cansino et al., 2002; Ranganath et al., 2004; Sommer et al., 2005; Staresina and Davachi, 2006; Uncapher et al., 2006; Kirwan et al., 2008; Park et al., 2008; Uncapher and Rugg, 2009; Gottlieb et al., 2010; Blumenfeld et al., 2011; Duarte et al., 2011; Song et al., 2011; Gottlieb et al., 2012; Rugg et al., 2012). As for associative memory encoding, the findings from these studies have consistently identified subsequent memory effects in the hippocampus and adjacent regions of the MTL [although see Kirwan et al. (2008) for an alternative interpretation of these findings]. As in the case of associative encoding (see above), the loci of cortical subsequent source memory effects have been reported to vary according to the nature of the contextual feature that was successfully encoded (e.g., Uncapher et al., 2006; Uncapher and Rugg, 2009; Gottlieb et al., 2010, 2012; Duarte et al., 2011). Unlike in the case of associative encoding, however, when subsequent memory effects in the LIFG are near-ubiquitous, subsequent source memory effects in this region are reported inconsistently. For example, in several studies, no LIFG effects were identified (Sommer et al., 2005; Kirwan et al., 2008; Park et al., 2008; Uncapher and Rugg, 2009; Gottlieb et al., 2010, 2012; Song et al., 2011), and LIFG effects were identified in only relatively small clusters (<20 voxels) in other studies (Cansino et al., 2002; Uncapher et al., 2006; Duarte et al., 2011). It is worth noting that the apparent failure of the LIFG to demonstrate robust subsequent source memory effects was evident not only when the encoding of the relevant contextual information was incidental (e.g., Sommer et al., 2005; Kirwan et al., 2008; Gottlieb et al., 2012), but also when subjects were informed before the study phase that their memory for the information would later be tested (e.g., Uncapher et al., 2006; Park et al., 2008).

In summary, prior fMRI studies have investigated the encoding of both associative and source memories. Consistent with a wealth of evidence implicating the MTL, and especially the hippocampus, in the encoding of arbitrary associations (Brown and Aggleton, 2001; Eichenbaum et al., 2007), encoding of both types of memory has consistently been associated with subsequent memory effects in this region. The two lines of research also converge to suggest that subsequent memory effects in neocortical regions outside of the MTL vary according to the nature of the information that is successfully encoded. A potential point of divergence, however, concerns the LIFG. As was noted above, whereas subsequent memory effects in this region are robust and extensive for the encoding of item-item associations across a variety of study materials and tasks, they seem to be much less in evidence when item-context associations are encoded. As we discuss later (see Discussion), this dissociation is consistent with proposals that the LIFG supports retrieval of, and selection between, competing representations of the different components of a study event.

Although suggestive, the comparison of findings across different studies does not establish that subsequent memory effects in the LIFG are greater for associative than for source encoding. Whether this is indeed the case requires studies in which the two types of subsequent memory effect are obtained from a common study task and are directly contrasted. To our knowledge, only one such study has been reported. In Park et al. (2012), subjects encoded picture pairs while concurrently hearing the pictures' names spoken in either a male or a female voice. They subsequently undertook an associative recognition test and, in addition, were required to recall the gender of the voice that had accompanied recognized test pairs during study. Subsequent memory effects in the LIFG were evident for the encoding of item-item, but not item-context associations.

Although the results for the LIFG reported by Park et al. (2012) are consistent with prior findings for associative and source encoding (see above), their interpretation is subject to two important caveats. First, there was a marked disparity in performance on the two memory tests. Whereas associative recognition performance was moderately high, source memory was almost at chance. Thus, the failure to identify LIFG subsequent source memory effects might merely have been a reflection of weak memory rather than an indication of a differential role for the region in associative vs. source encoding. The second caveat involves a possible attentional confound. As is typical in studies of associative encoding, the study task required that the members of each study pair were explicitly identified and relationally processed. By contrast, the source (voice) information was not incorporated into the study task, and hence did not need to be attended. It is therefore possible that the differential LIFG subsequent memory effects reported by Park et al. (2012) for associative and source encoding is a reflection of this attentional confound.

The aim of the present study was to directly compare the subsequent memory effects that accompany the encoding of item-item and item-context associations. We employed an experimental procedure in which memory for the two classes of association could be independently assessed from a series of formally identical study trials. Importantly, we employed a study task that ensured subjects directed their attention not only to the study items, but also to task-relevant contextual information. We focused on two primary questions: could any regions be identified where subsequent memory effects were common to the two classes of association? And where, if at all, would subsequent memory effects be found that were selective for associative or source encoding? We expected that the hippocampus would be among the regions to demonstrate a common subsequent memory effect (cf. Park et al., 2012). On the basis of the findings reviewed above, we also expected that the LIFG would be among the regions to show a selective effect, demonstrating greater and more extensive subsequent memory effects for associative than source encoding.

A second, more exploratory aim of the present study concerns so-called “negative” subsequent memory effects—effects that take the form of relatively lower activity for later remembered than later forgotten study events (see Kim, 2011 and Uncapher and Wagner, 2009 for reviews). Whereas robust negative effects have been reported for the encoding of item-item associations in several studies (e.g., Daselaar et al., 2004; Park and Rugg, 2008; de Chastelaine et al., 2011; Huijbers et al., 2012), to our knowledge there have been only two prior reports of such effects in relation to the encoding of item-context associations (Duarte et al., 2011; Gottlieb et al., 2012). Whether the scarcity of reports of negative subsequent source memory effects is because these effects are weaker and less reliable than in the case of associative encoding, or whether it merely reflects a reporting bias, is not clear. The present study affords the opportunity to address this issue by directly contrasting negative subsequent memory effects according to the type of association that is encoded.

## Materials and methods

### Subjects

Twenty-six volunteers (12 female; age range: 18–30 years, mean = 24, *SD* = 4.1) consented to participate in the study. All volunteers reported themselves to be right-handed fluent English speakers in good general health, with no history of neurological disease or other contraindications for MR imaging. Volunteers were recruited from local academic communities and were remunerated for their participation in accordance with the human subjects procedures approved by the Institutional Review Board of UTSW. Three volunteers' data were excluded because there were fewer than 10 trials in one or more critical experimental conditions. An additional three volunteers' data were excluded because of inadequate memory performance (>2 SDs below the sample mean for item recognition accuracy). Data are reported from the remaining 20 subjects (10 female; age range: 18–30 years, mean = 24, *SD* = 3.8).

### Stimulus materials

The critical experimental items consisted of 280 color pictures of common objects, each paired with a concrete noun. There was no overlap in the objects denoted by the pictures and the words. The pairings, which were consistent across subjects, were made on a pseudo-random basis under the constraints that each picture-word combination was semantically unrelated, and that for half of the pairs the object denoted by the word was larger than that denoted by the picture, and vice-versa for the remaining pairs. An additional 32 picture-word pairs were employed as filler or practice items. Pictures were selected from the Hemera Photo Objects 50,000 Volume II (Hemera Technologies Inc.), and the words were selected from the word association norms compiled by Nelson et al. (2004).

Study and test lists were separately generated for each subject. The type of trial to which each item pair was assigned (i.e., whether studied or unstudied, employed for the associative or source judgment, and study location), and the order in which the pairs were presented, varied randomly across subjects.

A study list comprised 200 randomly selected critical picture-word pairs, along with 12 filler pairs and 66 null trials. For each subject, the study list was broken down into three sublists (one per scan session), each of which contained 67 (66 for the third session) critical study pairs interspersed with 22 null trials. There was a 30 s rest break after the 47th trial in each session. Two filler trials were employed to buffer the beginning of each scan session and rest break.

**Figure 1:**
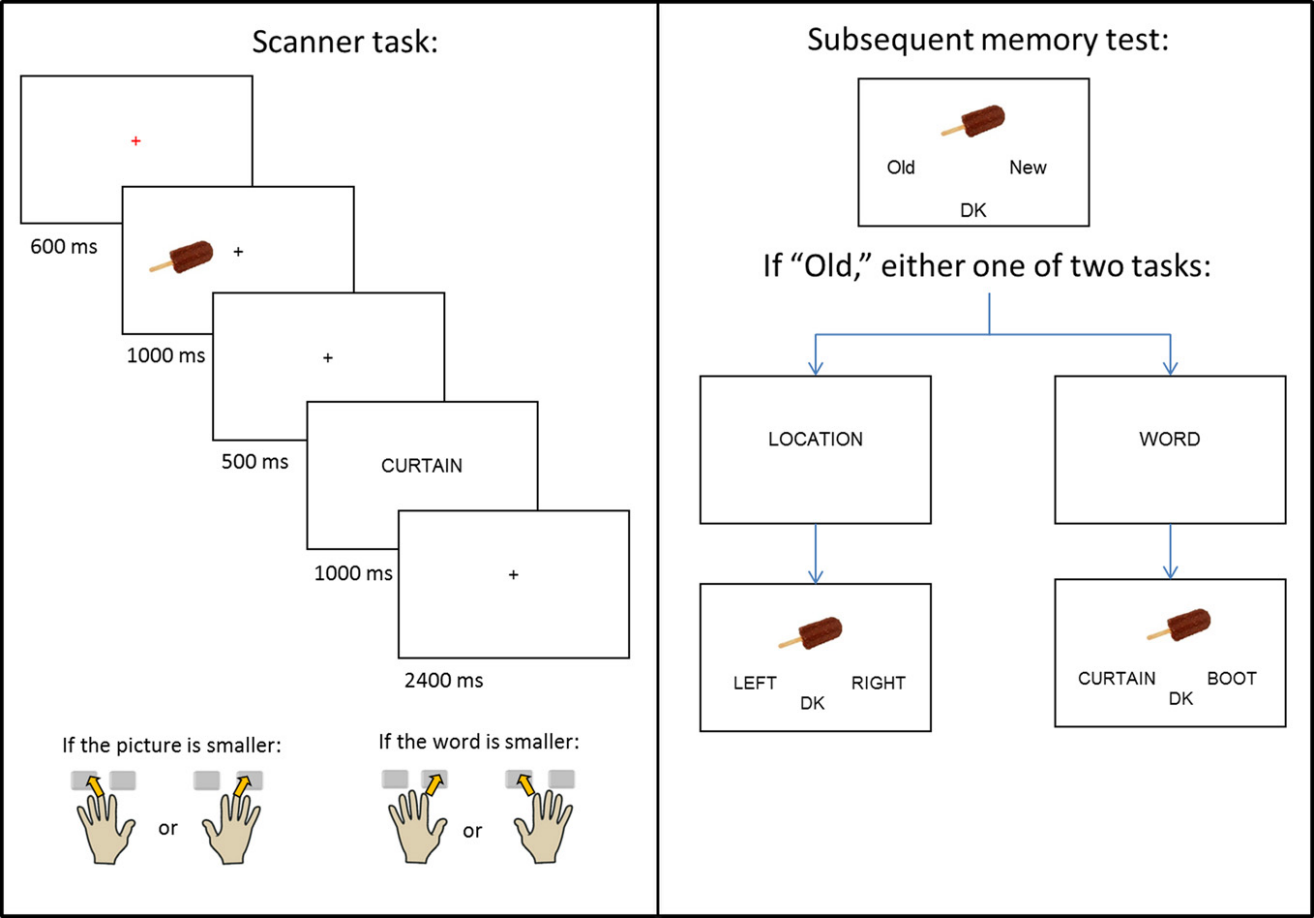
**Schematic of the experimental design**.

A test list consisted of 280 pictures, along with two filler pictures to buffer the beginning of the list. Two hundred of the pictures had been studied as picture-word pairs, and were randomly intermixed with 80 unstudied pictures. Each studied picture, if judged old, was co-presented with two previously studied words (one of which was the picture's pair-mate), or the words, “LEFT” and “RIGHT” (Figure 1, right panel). When an unstudied picture was judged old, it went on to be paired either with two unstudied words, or with the LEFT/RIGHT prompts. Thus, subjects received no feedback as to the accuracy of their judgments during the test. Because only half of the studied pictures were probed for their word associates, the lure words for the associative trials could be drawn without replacement from those study trials later tested for location. Thus, each studied word was presented only once at test.

### Procedure

The experiment consisted of a single study-test cycle. Instructions and practice for the study task were given outside the scanner, with the exception of one short practice given in the scanner to orient subjects to the use of the button boxes. A schematic of the study design is given in Figure 1. During study trials, subjects viewed pictures, presented either to the left or right of central fixation, which were followed by a word presented at the center of the screen. The beginning of each trial was indicated by a red central fixation cross (“+”) presented for 600 ms. The picture then appeared for 1000 ms, before being replaced by another black cross. After 500 ms, the cross was replaced by the word for 1000 ms, which was replaced by a final black cross lasting for 2400 ms.

The presentation of the picture served as a signal for subjects to prepare to use the hand that corresponded to the picture's location (left or right) on the screen. When the word appeared, subjects used either their left or right hand (depending on the location of the picture) to indicate whether the object denoted by the picture was smaller (middle finger), or larger (index finger) than the object denoted by the word. Pictures appeared with equal frequency at each location, and no more than three consecutive pictures were presented at the same location. Each study session consisted of 67 (66 for the third session) picture-word pairs, a 30 s break after the 47th trial, and four buffer trials. Inter-trial interval was stochastically distributed with a minimum interval of 5.5 s modulated by the addition of approximately one-quarter (22) randomly intermixed null trials (Josephs and Henson, 1999).

A non-scanned memory test (Figure 1, right panel) was administered 20 min after the end of the final study session. The memory test took place outside of the scanner, and the study-test interval was filled by conversation with the experimenter. All 200 critical study pictures were presented, one at a time, along with 80 randomly interspersed unstudied (new) pictures. Subjects were instructed to judge whether each picture was old or new and to indicate their decision using the index fingers of their left or right hand. If unsure, they were instructed to choose a third, “don't know” (DK) option. For pictures that were judged old, either an associative or source memory judgment was then required. In the former case, two previously studied words were presented below the picture, with the requirement to select the word previously paired with the picture at study. In the source task, the words “LEFT” and “RIGHT” were presented below the picture, with the requirement to select the study location of the picture. In both tasks, a “don't know” option was also available. Prior to each of the associative and source tasks, the words “WORD?” or “LOCATION?” appeared in the middle of the screen to prepare the subject for the judgment to be made. Of the pictures presented to the left of fixation at study, half appeared at test with the “WORD?” task and half with the “LOCATION?” task; the same distribution applied to pictures that appeared to the right of fixation at study. Trials testing for either associative or source memory were randomly ordered, with no more than three consecutive trials of the same type. Test trials were self-paced and presented as a single block.

### fMRI scanning

A Philips Achieva 3T MR scanner (Philips Medical Systems, Andover, MA, USA) equipped with a 32 channel head coil was used to acquire both T1-weighted anatomical images (256 × 224 matrix, 1 mm^3^ voxels, 160 slices, sagittal acquisition) and T2^*^-weighted echoplanar images (SENSE factor of 1.5, flip angle 70°, 80 × 78 matrix, FOV = 24 cm, *TR* = 2000 ms, *TE* = 30 ms). Each volume comprised 33 slices oriented parallel to the anterior-posterior commissure plane (3-mm thick slice, 1 mm interslice gap, 3 mm isotropic voxels) and was acquired in an ascending sequence. Data were acquired during the study phase in three scanning sessions, with the first two sessions comprising 280 volumes and the last comprising 277 volumes. The 5.5 s SOA allowed for an effective sampling rate of the hemodynamic response of 2 Hz. The first five volumes of each session were discarded to allow equilibration of tissue magnetization.

### fMRI data analysis

Data preprocessing and analyses were performed with Statistical Parametric Mapping (SPM8, Wellcome Department of Cognitive Neurology, London, UK), implemented in MATLAB 2008 (The Mathworks, Inc., USA). Functional images were subjected to a two-pass spatial realignment. Images were realigned to the first image, generating a mean image for each session. In the second pass, the raw images were then realigned to the session-specific mean. Correction for differences in acquisition times was performed by sinc interpolation with respect to the acquisition time of the middle slice in each volume. The images were then subjected to reorientation, spatial normalization to a standard EPI template [based on the Montreal Neurological Institute (MNI) brain; Cocosco et al., 1997] and smoothing with an 8 mm FWHM Gaussian kernel. Because of the relatively small numbers of events of interest present in each separate scanning session, functional time series were concatenated across sessions.

For each subject, study activity was modeled by a 5 s duration boxcar function that began with picture onset on each study trial. The predicted blood oxygen level dependent (BOLD) response was modeled by convolving these neural functions with a canonical hemodynamic response function. The great majority of subjects had too few trials in the “associative DK” (mean = 4.6, *SD* = 4.7) and “source DK” (mean = 3.1, *SD* = 3.1) response categories to allow for stable estimates of the associated neural activity. Therefore, these DK trials were collapsed with the associative incorrect and source incorrect trials, respectively. The principal analyses were confined to four events of interest: trials on which subjects remembered the word associated with a recognized picture (associative correct), trials for which the picture was recognized but the associated word was not remembered (associative incorrect), trials on which subjects remembered the location associated with a recognized picture (source correct), and recognized pictures for which the location was not remembered (source incorrect). Item misses were modeled as a separate category. A final category comprised events of no interest, and included filler trials, null trials, and trials associated with multiple or missed button presses. In addition, six regressors were employed to model movement-related variance, and session-specific constant terms were employed to model mean image intensity in each of the three sessions.

The functional timeseries was highpass-filtered to 1/128 Hz and scaled within-session to yield a grand mean of 100 across voxels and scans (the default settings within SPM; http://www.fil.ion.ucl.ac.uk/spm/doc/manual.pdf). Parameter estimates for events of interest were estimated using a General Linear Model. Nonsphericity of the error covariance was accommodated by an AR(1) model, in which the temporal autocorrelation was estimated by pooling over suprathreshold voxels (Friston et al., 2002). The parameters for each covariate and the hyperparameters governing the error covariance were estimated using Restricted Maximum Likelihood (ReML). Parameter estimates for the four conditions of interest (associative correct, associative incorrect, source correct, and source incorrect) were derived for each subject and carried forward to a second level group-wise analysis. In this analysis, individual subjects' parameter estimates for the four conditions of interest were entered into a repeated-measures one-way ANOVA model, as implemented in SPM8. Planned contrasts assessing the different effects of interest were performed using the common error term derived from the ANOVA. Protection against Type I error was effected by using the “Analysis of Functional Neuroimages” (AFNI) AlphaSim tool (http://afni.nimh.nih.gov/afni/AFNI_Help/AlphaSim.html) to estimate the minimum cluster size necessary for a cluster-wise corrected significance level of *p* < 0.05 at a height-threshold of *p* < 0.005. The critical value was 47 contiguous voxels. As described in the Results section, each planned contrast was inclusively masked with additional contrast(s) to identify subsequent memory effects selective for, or common to, associative or source encoding. The 47 voxel extent threshold was maintained after application of the masks. In light of our pre-experimental prediction that both source and associative subsequent memory effects would be identified in the MTL, AlphaSim was also used to estimate the voxel extent threshold within a mask restricted to the bilateral MTL. The critical value, for a corrected significance level of *p* < 0.05 at a height-threshold of *p* < 0.005, was 17 voxels.

## Results

### Behavioral performance

#### Study task

Mean accuracy on the study task was 0.83 (*SD* = 0.06). Study RTs are shown in Table 1 segregated according to later memory performance. A one-way ANOVA revealed no significant differences in RT between the four subsequent memory conditions (*F* < 1).

**Table 1:** **Mean reaction times (ms) for correct size/hand judgments at study segregated by subsequent memory (SD in parentheses)**.

**Subsequent memory judgment**	**Reaction time (ms)**
Associative correct	1509 (439)
Associative incorrect/DK	1496 (441)
Source correct	1504 (457)
Source incorrect/DK	1460 (410)

#### Retrieval task

The item hit rate was 0.81 (*SD* = 0.10) against a false alarm rate of 0.16. Conditionalized on accurate item recognition, the proportions of accurate associative and source judgments were 0.68 (*SD* = 0.07) and 0.74 (*SD* = 0.11) respectively. Following prior studies (e.g., Gottlieb et al., 2012), associative and source memory were estimated with an index derived from a single high-threshold model, in which the probability of recollection was computed as *p*_(recollection)_ = {*p*_(Hits)_ − 0.5[1 − *p*_(DK)_]}/{1 − 0.5[1 − *p*_(DK)_]}. The index was calculated separately for associative and source memory. Associative and source memory estimates were 0.40 (*SD* = 0.11) and 0.51 (*SD* = 0.20), respectively. Pairwise contrasts revealed that both performance estimates were significantly greater than the chance value of zero [*t*_(19)_ = 16.86, *p* < 0.001, and *t*_(19)_ = 11.39, *p* < 0.001 for associative and source judgments, respectively], and that the associative memory judgments were significantly less accurate than the source memory judgments [*t*_(19)_ = 2.35, *p* < 0.05]. The correlation between performance on the two memory tests was low and not significant (*r* = 0.23, *p* = 0.33).

### fMRI results

**Figure 2:**
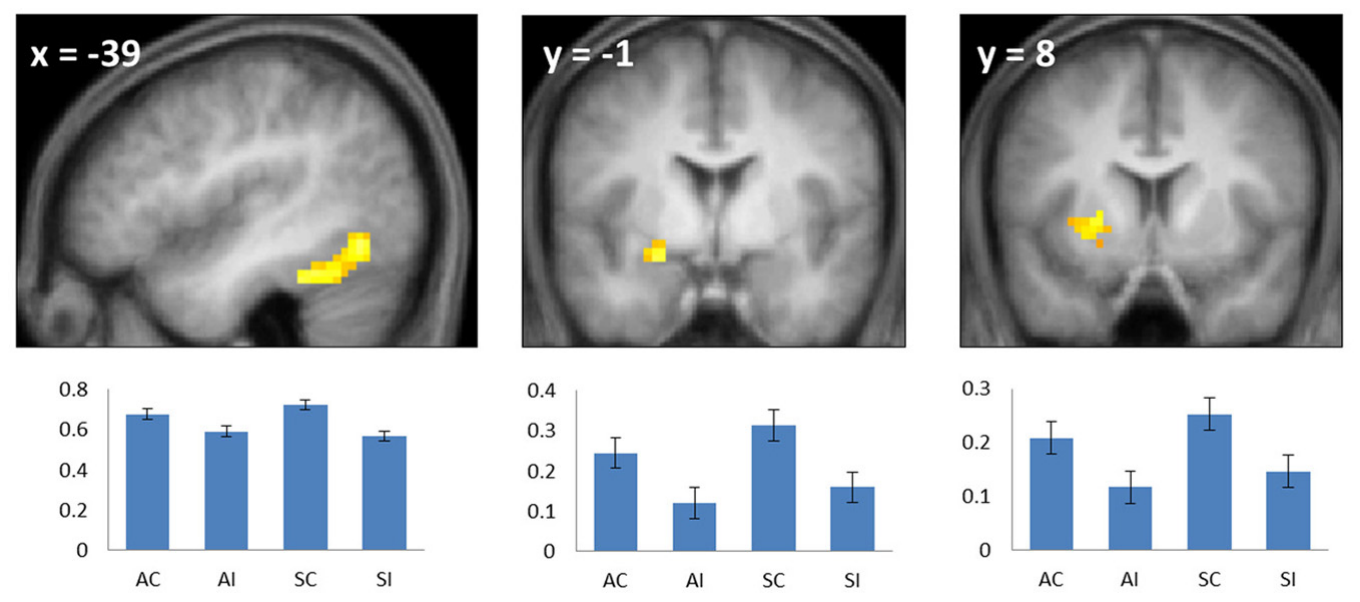
**Common subsequent memory effects.** Bar plots show mean parameter estimates (left to right) for the four conditions of interest (AC, associative correct; AI, associative incorrect; SC, source correct; SI, source incorrect) for peak voxels in the left fusiform (left), left anterior hippocampus/amygdala (center), and left putamen (right). Results are overlaid onto sections of the across-subjects mean T1-weighted anatomical image (note—in this and subsequent figures, the mean image is derived from only 19 of the 20 included subjects, because of the corruption of one subject's anatomical data). Error bars here and in the following figures signify the standard error of the mean derived from the error term of the one-way ANOVA (Loftus and Masson, 1994).

**Figure 3:**
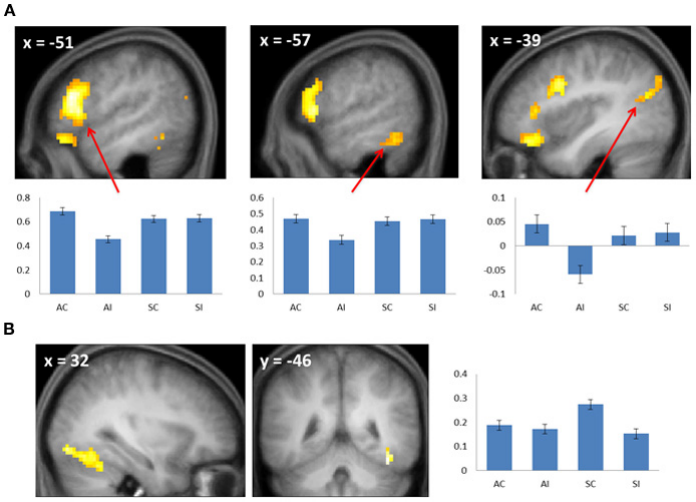
**(A)** Subsequent associative memory effects. Bar plots show (left to right) mean parameter estimates for the four conditions of interest for peak voxels (arrows) in LIFG, left inferior temporal gyrus, and left angular gyrus. **(B)** Subsequent source memory effect in the right fusiform cortex. Two views of the same cluster are shown. Bar plot shows the peak parameter estimates for the four conditions of interest. Results are overlaid onto sections of the across-subjects mean T1-weighted anatomical image.

**Figure 4:**
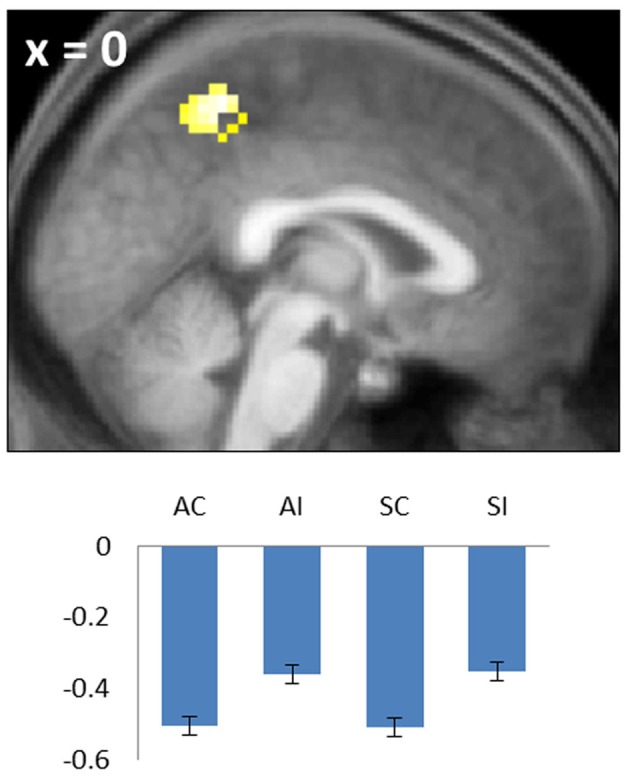
**Negative common subsequent memory effect in the medial parietal cortex (precuneus).** Bar plot shows the peak parameter estimates for the four conditions of interest. Results are overlaid onto a sagittal section of the across-subjects mean T1-weighted anatomical image.

**Figure 5:**
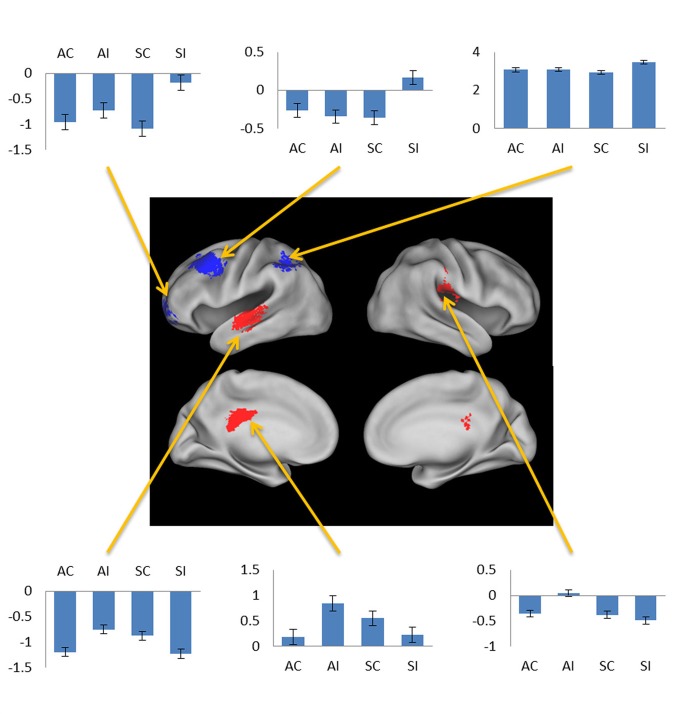
**Negative subsequent memory effects selective for associative (red) and source (blue).** Bar plots show mean parameter estimates for the four conditions of interest for peak voxels (arrows, clockwise) in left orbitofrontal gyrus, left middle frontal gyrus, left intraparietal sulcus, right posterior insula, left posterior cingulate gyrus, and left superior temporal gyrus. Results are overlaid on the standardized brain of the PALS-B12 atlas implemented in Caret5 (Van Essen, 2005).

Regions demonstrating subsequent memory effects were identified using an ANOVA model (levels of associative correct, associative incorrect, source correct, and source incorrect). We first identified subsequent memory effects that were common to the two classes of association. We then identified effects that were selective for each class. For illustrative purposes only (Kriegeskorte et al., 2009), parameter estimates for source and associative effects are shown in Figures 2–5.

#### Common subsequent memory effects

To identify regions demonstrating subsequent memory effects common across associative and source recollection, the main effect of recollection (associative correct + source correct > associative incorrect + source incorrect; thresholded at *p* < 0.005) was inclusively masked with the separate subsequent memory contrasts for associative and source memory (associative correct > associative incorrect and source correct > source incorrect, respectively; each thresholded at *p* < 0.05 one-sided). Thus, the resulting SPM identified voxels where the main effect was accompanied by reliable simple effects for the constituent subsequent memory contrasts (cf. Park and Rugg, 2011; Gottlieb et al., 2012). Common effects were identified in left fusiform cortex, the left anterior hippocampus/amygdala, and the left putamen (Figure 2; Table 2). The main effect of recollection alone (i.e., not masked with the respective simple effects) included an additional cluster in left hippocampus (peak at −30, −22, −20; *Z* = 3.0; 22 voxels).

**Table 2:** **Loci of subsequent memory effects**.

**Region**	**Coordinates**			**Peak *Z***	**Number of above-threshold voxels**
	***x***	***y***	***z***		
**COMMON EFFECTS**					
L amygdala/anterior hippocampus	−24	−1	−14	3.85	65
L putamen (subpeak)	−21	8	1	3.67	
L fusiform cortex	−39	−52	−20	4.87	152
**SELECTIVE ASSOCIATIVE MEMORY EFFECTS**					
L orbitofrontal cortex	−36	35	−17	4.16	155
L inferior frontal gyrus	−51	26	22	4.99	481
L superior frontal sulcus	−24	23	49	3.24	48
L inferior temporal gyrus	−57	−52	−17	3.25	47
L angular gyrus	−39	−70	22	3.55	90
R cerebellum	12	−82	−35	3.23	48
**SELECTIVE SOURCE MEMORY EFFECTS**					
R fusiform cortex	42	−46	−17	4.21	167
**COMMON NEGATIVE EFFECTS**					
Medial parietal cortex (precuneus)	0	−46	58	3.84	125

#### Selective subsequent memory effects

Subsequent memory effects selective for associative memory were identified by inclusively masking the relevant subsequent memory contrast (associative correct > associative incorrect; thresholded at *p* < 0.005) with the directional interaction contrast that identified voxels where associative effects exceeded source effects [(associative correct > associative incorrect) > (source correct > source incorrect); thresholded at *p* < 0.05]. Thus, the resulting SPM identified voxels that demonstrated a reliable subsequent associative memory effect that was also reliably greater than the corresponding source memory effect. The procedure identified effects throughout the extent of the LIFG, along with other regions documented in Figure 3A and Table 2. The analogous pair of contrasts was performed to identify effects selectively associated with the encoding of source information. These identified a single cluster in right fusiform cortex (Figure 3B; Table 2).

#### Negative subsequent memory effects

Negative subsequent memory effects were identified by performing the reverse of the contrasts described above. Thus, common effects were identified by inclusively masking the relevant main effect (associative correct + source correct) < (associative incorrect + source incorrect) with the constituent pairwise contrasts (associative correct < associative incorrect and source correct < source incorrect; each thresholded at *p* < 0.05). The resulting SPM revealed a single cluster in medial parietal cortex (precuneus; see Table 2; Figure 4). No negative effects selective for either associative or source memory were identified.

#### Additional analyses

We employed three additional analysis models to investigate the generality of the findings reported above.^1^ The first model was identical to that employed in the primary analysis except that study trials associated with associative or source “don't know” responses were modeled as events of no interest, rather than being pooled with the trials given an incorrect response (one subject was eliminated from this analysis because of too few source incorrect trials). The results were somewhat weaker (cluster sizes were reduced relative to the original analyses for the common positive and negative subsequent memory effects, and for the selective source memory effect), but qualitatively very similar, to those reported above.

The other two models addressed the question of whether the employment of a single boxcar regressor in the primary analyses obscured effects selectively associated with picture or word onset. Accordingly, these two models employed as regressors delta functions (convolved with a canonical hemodynamic response function) that modeled the activity elicited by the onset of the picture or word, respectively. With two exceptions (see below) these models gave rise to effects that were a sub-set of those identified in the primary analyses described in the previous section and, in both cases, identified selective subsequent associative memory effects in the LIFG (indicating that the effects are not idiosyncratic to a specific model of trial-related activity). Importantly, in neither model did we identify subsequent memory effects additional to those identified in the main analysis.

The exceptions to this pattern concerned the negative subsequent memory effects captured by the regressor modeling activity elicited by the onset of the pictures. As is illustrated in Figure 5 and detailed in Table 3, this model identified above-threshold selective negative subsequent associative memory effects in bilateral temporal cortex, and selective negative source effects in left frontal and superior parietal cortex.

**Table 3:** **Loci of selective negative subsequent memory effects identified in subsidiary analyses**.

**Region**	**Coordinates**			**Peak *Z***	**Number of above-threshold voxels**
	***x***	***y***	***z***		
**SELECTIVE ASSOCIATIVE NEGATIVE EFFECTS**					
L superior temporal gyrus	−57	−13	−8	3.53	70
L posterior cingulate gyrus	−6	−37	25	3.12	49
R posterior insula	48	−19	25	4.02	91
**SELECTIVE SOURCE NEGATIVE EFFECTS**					
L orbitofrontal gyrus	−24	56	−5	3.74	56
L middle frontal gyrus	−30	20	43	3.66	117
L intraparietal sulcus	−33	−55	46	3.21	57

## Discussion

The present study directly compared the neural correlates of encoding item-item and item-context associations. We used a procedure where subjects studied pictures (presented in one of two spatial contexts) in association with words. Four types of subsequent memory effect were identified: effects common to associative and source encoding, effects selectively linked to the encoding one or other class of association, a common negative subsequent memory effect and, in a subsidiary analysis, selective negative subsequent memory effects.

### Behavioral findings

There were no significant RT differences between study trials according to performance on the later memory test, making it unlikely that any fMRI subsequent memory effects merely reflected differences in the efficiency with which the study events were processed. Source memory was more accurate than associative memory, raising the possibility that the larger subsequent memory effect in the right fusiform that was identified for source relative to associative encoding (Figure 3B) may merely have reflected a difference in memory strength. Crucially, since associative memory was weaker than source memory, this potential confound cannot be responsible for the finding that several regions, including the LIFG, demonstrated subsequent memory effects that were selective for associative encoding.

There was a weak and non-significant correlation across subjects between associative and source memory performance. This finding suggests that the processes supporting the encoding of item-item and item-context associations were at least partially independent. This is consistent with the findings, discussed below, indicating that subsequent associative and source memory effects are anatomically dissociable.

### fMRI findings

#### Common effects

Subsequent memory effects common to both types of association were identified in left fusiform cortex, the putamen, the left anterior MTL on the border between the hippocampus and amygdala and, when a less stringent criterion was applied, in the body of the left hippocampus. The findings for the anterior MTL and hippocampus are consistent with numerous prior reports of subsequent source and associative effects in these regions (e.g., Jackson and Schacter, 2004; Summerfield et al., 2006; Chua et al., 2007; Gottlieb et al., 2012 see Kim, 2011 for review). The common effect evident in left fusiform cortex likewise replicate prior findings, with respect to both source (e.g., Gottlieb et al., 2012) and associative (e.g., Chua et al., 2007; Park and Rugg, 2011) encoding (see Kim, 2011, for review).

We also identified a common subsequent memory effect in the putamen, consistent with the findings of several prior studies that reported subsequent memory effects both in the putamen and other striatal regions during episodic encoding (e.g., Sperling et al., 2003; Prince et al., 2005; Adcock et al., 2006; Park and Rugg, 2011; Gottlieb et al., 2012). It has been hypothesized (Sadeh et al., 2011) that the contribution of the striatum to episodic encoding is through its role in controlling the contents of working memory (McNab and Klingberg, 2008). In the current study, the requirements to remember which hand to use for the study judgment, as well as the identity of the study picture, likely taxed working memory. Thus, it is possible that the common subsequent memory effect in the putamen reflects the benefit to encoding that occurred when these two components of the study event were represented in working memory to the exclusion of task-irrelevant information.

#### Selective effects

The most important finding in the present study involves the selectivity of the LIFG for the encoding of associative rather than source information: whereas subsequent associative memory effects in this region were robust and extensive, they were essentially undetectable for the encoding of source information (even at a *p* < 0.05 threshold, the source correct > source incorrect contrast identified only 2 voxels that overlapped with the LIFG subsequent associative memory effect). This finding is consistent with the impression gained from the prior literature (see Introduction), and with the findings of a prior study that also directly contrasted subsequent associative and source memory effects (Park et al., 2012). Unlike in that study, however, the present finding cannot be a consequence of weaker memory for source than associative information (see above), and nor can it be attributed to the absence of a requirement to explicitly attend to relevant contextual information (see Introduction).

A reviewer suggested that our finding that LIFG subsequent memory effects were selective for successful associative encoding may reflect weaker lateralization of subsequent memory effects in the inferior frontal gyrus for source than for associative encoding. By this argument, variable or weak lateralization of the processes supporting source encoding could make the effects difficult to detect, especially if, as in the present study, subjects were not assessed for strength of lateralization of function beyond self-reported handedness. While it is not possible to conclusively reject this proposal, the finding that LIFG subsequent source memory effects were essentially undetectable (see above) is consistent with the view that this region did indeed play little or no role in the encoding of item-context associations in the present study.

What light do the current findings shed on the role of the LIFG in episodic memory encoding? It has been proposed that this region supports such functions as the controlled retrieval of semantic (and other) representations, and the selection among competing representations of the one most appropriate for the current cognitive context (e.g., Thompson-Schill et al., 1997, 1999; Gold and Buckner, 2002; Badre and Wagner, 2007). These functions are likely to be engaged in study tasks typically employed in studies of associative (item-item) encoding [e.g., whether a name provided a good fit to a face (Sperling et al., 2003); generating a “mental image” incorporating both items (Jackson and Schacter, 2004); or judging which item would “fit” inside the other in (Park and Rugg, 2011)]. From this perspective, subsequent associative memory effects in the LIFG can be understood in terms of the principle that cortical subsequent memory effects reflect modulation of activity in regions engaged during the on-line processing of a study event (Rugg et al., 2008). For example, one possibility is that relative enhancement of LIFG activity during successful associative encoding supports the generation of well-specified representations of the two study items and their task-relevant attributes, and that such representations are especially conducive to the formation of a durable associative memory.

This is not to say that the LIFG cannot also be engaged during the processing of individual study items. Indeed, subsequent memory effects in this region have been reported for the encoding of individual items since the inception of the fMRI subsequent memory procedure (Wagner et al., 1998), and are particularly prominent when items are subjected to semantically-oriented study (e.g., Otten and Rugg, 2001). Why, then, is LIFG activity not enhanced during successful source encoding, when a single item must be associated with a contextual feature (rather than another study item, as in associative encoding)? We conjecture that the absence of LIFG subsequent source memory effects reflects the fact that the formation of an item-context association usually does not require controlled retrieval and selection beyond what is needed to generate a task-appropriate representation of the item itself: typically, contextual information (for example, spatial location, color, or sensory modality) is in the form of perceptual features, the representation of which is largely bottom-up, with little need for selection among competitors. In short, whereas the processing of two items engages the LIFG to a greater extent than does the processing of a single item, the processing of an item in association with one or more contextual features typically does not.

It might be put forth that the foregoing account can be reduced to the argument that LIFG subsequent memory effects merely reflect the level of processing to which a given component of the study event was subjected. By this argument the effects are prominent when a component is “deeply” processed (i.e., to the level of its meaning), but not when it is processed more superficially. Other findings suggest however that this is unlikely to be a sufficient explanation for the dissociation in this region between subsequent associative and source memory effects. Park and Rugg (2008) contrasted subsequent associative memory effects according to whether the study pairs were judged for their semantic or phonological similarity (a typical depth of processing manipulation; Craik and Lockhart, 1972). As would be expected, subsequent memory performance was markedly better for the pairs from the semantic task. Nonetheless, the robust subsequent memory effects identified in the LIFG did not differ in magnitude as a function of study task (and, hence, depth of processing). These findings can be accommodated by the foregoing account if it assumed that both study tasks required resolution between potentially competing representations of each member of the study pairs (semantic representations in one case and phonological in the other).

If the foregoing account is correct, the question arises as to why subsequent source memory effects in the LIFG have sometimes been reported (e.g., Ranganath et al., 2004; Staresina and Davachi, 2006; Blumenfeld et al., 2011; Duarte et al., 2011). One possibility of course is that processing of the source feature in these studies required engagement of the LIFG. Another possibility, however, is that the LIFG effects reflect a confound between the accuracy of source memory and strength of item memory: other things being equal, the accuracy and confidence with which test items are recognized is greater for items associated with a correct source judgment than it is for items associated with an incorrect judgment (e.g., Slotnick and Dodson, 2005; Kirwan et al., 2008; Song et al., 2011). Thus, as has been argued for subsequent source memory effects in the hippocampus (Kirwan et al., 2008; Song et al., 2011; but see Rugg et al., 2012), effects in the LIFG might reflect the differential role of this region in the encoding of relatively strong vs. relatively weak item memories rather than in the encoding of item-context associations.

A second region to demonstrate selective subsequent associative memory effects was ventral parietal cortex in the vicinity of the angular gyrus. Similar findings have been reported in prior studies of associative encoding (Park and Rugg, 2008; de Chastelaine et al., 2011). The angular gyrus is often held to belong to the default mode network, and typically exhibits negative rather than positive subsequent memory effects (Uncapher et al., 2011). The region is, however, heavily implicated in the processing of semantic and conceptual information (Binder and Desai, 2011; Jefferies, 2013). Furthermore, it has been reported that regions within the angular gyrus that demonstrate task-related deactivation (consistent with a role in default mode processing), and regions that are selectively active during semantic processing, are partially dissociable (Seghier et al., 2010). Therefore it seems likely that the subsequent associative memory effects identified in this region in the present and prior studies reflect the twin facts that the region was recruited in service of the demands of the study task, and that associative encoding benefited when processing of the semantic features of the items was emphasized.

The final region to demonstrate selective subsequent associative memory effects was in left inferior temporal cortex, lateral to the nearby common effect (see Table 2). It has been proposed that this region supports access to semantic knowledge of both words and objects (Jobard et al., 2003; Binder et al., 2009). Therefore, as in the case of the subsequent associative memory effect in the angular gyrus, the left inferior temporal effect may reflect the benefit to encoding that accrued when a relatively large amount of resources were allocated to semantic processing of the items.

In contrast to subsequent associative memory effects, selective source memory effects were confined to a single cluster in right fusiform cortex. This finding adds to prior reports of subsequent source memory effects for objects in right occipito-temporal cortex (e.g., Cansino et al., 2002; Ranganath et al., 2004; Sommer et al., 2005; Uncapher and Rugg, 2009). The present finding represents an advance over these previous reports in that they implicate the region in the encoding specifically of object-context associations, rather than associative encoding more generally. As was discussed by Gottlieb et al. (2012), it is unclear why enhanced activity in a brain region strongly implicated in object processing should facilitate the encoding of such associations. The present findings strengthen the evidence supporting a role for right fusiform cortex in the encoding of object-context associations, but do not further understanding of the underlying mechanism.

#### Negative subsequent memory effects

A robust negative common subsequent memory effect was identified in medial parietal cortex. The location of this effect is consistent with the findings of numerous prior studies in which similar effects were reported across a variety of study materials and tasks (Kim, 2011), including two prior studies of source encoding (Duarte et al., 2011; Gottlieb et al., 2012). Negative subsequent memory effects are thought to result mainly from modulation of “default mode” activity (Gusnard and Raichle, 2001; Buckner et al., 2008), reflecting the benefit to encoding that accrues when attentional resources are fully withdrawn from internally-directed cognition and allocated to the study event (Daselaar et al., 2004; Uncapher and Wagner, 2009). Together with prior findings, the present results suggest that disengagement of default processes is beneficial for the encoding of both item-item and item-context associations.

In addition to the common negative subsequent memory effect discussed above, a secondary analysis employing a regressor modeling activity elicited at picture onset identified selective associative and source effects. The finding that these effects were identified only in this analysis suggests that they reflect processes elicited by the pictures that differentially facilitated the incorporation of the pictures into item-item or item-context associations. The identity of these processes is currently obscure. Together with prior evidence for feature-selective negative subsequent memory effects (Gottlieb et al., 2012), the present findings do suggest however that negative subsequent memory effects reflect more than the modulation of generic processes—such as those supported by the “default-mode network”—that impact episodic encoding in a non-selective fashion.

## Concluding comments

The present study directly contrasted subsequent memory effects accompanying successful encoding of associative (item-item) and source (item-context) encoding. The findings indicate that the two classes of effect can be dissociated across different cortical regions, suggesting that the encoding of source and associative information depend on partially independent neural mechanisms. The findings further suggest that the LIFG plays a markedly more important role in associative than in source encoding.

### Conflict of interest statement

The authors declare that the research was conducted in the absence of any commercial or financial relationships that could be construed as a potential conflict of interest.

## Footnotes

^1^We thank two anonymous reviewers for suggesting these additional analyses.

## References

[B1] Adcock R. A. Thangavel A. Whitfield-Gabrieli S. Knutson B. Gabrieli J. D. E. (2006). Reward-motivated learning: mesolimbic activation precedes memory formation. Neuron 50, 507–517 10.1016/j.neuron.2006.03.036 16675403

[B2] Badre D. Wagner A. D. (2007). Left ventrolateral prefrontal cortex and the cognitive control of memory. Neuropsychologia 45, 2883–2901 10.1016/j.neuropsychologia.2007.06.015 17675110

[B3] Binder J. R. Desai R. H. (2011). The neurobiology of semantic memory. Trends Cogn. Sci. 15, 527–536 10.1016/j.tics.2011.10.001 22001867 PMC3350748

[B4] Binder J. R. Desai R. H. Graves W. W. Conant L. L. (2009). Where is the semantic system? A critical review and meta-analysis of 120 functional neuroimaging studies. Cereb. Cortex 19, 2767–2796 10.1093/cercor/bhp055 19329570 PMC2774390

[B5] Blumenfeld R. S. Parks C. M. Yonelinas A. P. Ranganath C. (2011). Putting the pieces together: the role of dorsolateral prefrontal cortex in relational memory encoding. J. Cogn. Neurosci. 23, 257–265 10.1162/jocn.2010.21459 20146616 PMC3970078

[B6] Brown M. W. Aggleton J. P. (2001). Recognition memory: what are the roles of the perirhinal cortex and hippocampus? Nat. Rev. Neurosci. 2, 51–61 11253359 10.1038/35049064

[B7] Buckner R. L. Andrews-Hanna J. R. Schacter D. L. (2008). The brain's default network: anatomy, function, and relevance to disease. Ann. N.Y. Acad. Sci. 1124, 1–38 10.1196/annals.1440.011 18400922

[B8] Cansino S. Maquet P. Dolan R. J. Rugg M. D. (2002). Brain activity underlying encoding and retrieval of source memory. Cereb. Cortex 12, 1048–1056 10.1093/cercor/12.10.1048 12217968

[B9] Chua E. F. Schacter D. L. Rand-Giovannetti E. Sperling R. A. (2007). Evidence for a specific role of the anterior hippocampal region in successful associative encoding. Hippocampus 17, 1071–1080 10.1002/hipo.20340 17604351

[B10] Cocosco C. Kollokian V. Kwan R. K. S. Pike G. Evans A. (1997). BrainWeb: online interface to a 3D MRI simulated brain database. Neuroimage 5, S425

[B11] Craik F. I. M. Lockhart R. S. (1972). Levels of Processing: a framework for memory research. J. Verb. Learn. Verb. Behav. 11, 671–684 10.1016/S0022-5371(72)80001-X

[B12] Daselaar S. M. Prince S. E. Cabeza R. (2004). When less means more: deactivations during encoding that predict subsequent memory. Neuroimage 23, 921–927 10.1016/j.neuroimage.2004.07.031 15528092

[B14] de Chastelaine M. Wang T. H. Minton B. Muftuler L. T. Rugg M. D. (2011). The effects of age, memory performance, and callosal integrity on the neural correlates of successful associative encoding. Cereb. cortex 21, 2166–2176 10.1093/cercor/bhq294 21282317 PMC3155606

[B15] Duarte A. Henson R. N. Graham K. S. (2011). Stimulus content and the neural correlates of source memory. Brain Res. 1373, 110–123 10.1016/j.brainres.2010.11.086 21145314 PMC3098368

[B16] Eichenbaum H. Yonelinas A. P. Ranganath C. (2007). The medial temporal lobe and recognition memory. Annu. Rev. Neurosci. 30, 123–152 10.1146/annurev.neuro.30.051606.094328 17417939 PMC2064941

[B17] Friston K. J. Penny W. Phillips C. Kiebel S. Hinton G. Ashburner J. (2002). Classical and Bayesian inference in neuroimaging: theory. Neuroimage 16, 465–483 10.1006/nimg.2002.1090 12030832

[B18] Gold B. T. Buckner R. L. (2002). Common prefrontal regions coactivate with dissociable posterior regions during controlled semantic and phonological tasks. Neuron 35, 803–812 10.1016/S0896-6273(02)00800-0 12194878

[B19] Gottlieb L. J. Uncapher M. R. Rugg M. D. (2010). Dissociation of the neural correlates of visual and auditory contextual encoding. Neuropsychologia 48, 137–144 10.1016/j.neuropsychologia.2009.08.019 19720071 PMC2795095

[B20] Gottlieb L. J. Wong J. de Chastelaine M. Rugg M. D. (2012). Neural correlates of the encoding of multimodal contextual features. Learn. Mem. 19, 605–614 10.1101/lm.027631.112 23166292 PMC3506975

[B21] Gusnard D. A. Raichle M. E. (2001). Searching for a baseline: functional imaging and the resting human brain. Nat. Rev. Neurosci. 2, 685–694 10.1038/35094500 11584306

[B22] Huijbers W. Vannini P. Sperling R. A. Pennartz C. M. Cabeza R. Daselaar S. M. (2012). Explaining the encoding/retrieval flip: memory-related deactivations and activations in the posteromedial cortex. Neuropsychologia 50, 3764–3774 10.1016/j.neuropsychologia.2012.08.021 22982484 PMC3811140

[B23] Jackson O. Schacter D. L. (2004). Encoding activity in anterior medial temporal lobe supports subsequent associative recognition. Neuroimage 21, 456–462 10.1016/j.neuroimage.2003.09.050 14741683

[B24] Jefferies E. (2013). The neural basis of semantic cognition: converging evidence from neuropsychology, neuroimaging and TMS. Cortex 49, 611–625 10.1016/j.cortex.2012.10.008 23260615

[B25] Jobard G. Crivello F. Tzourio-Mazoyer N. (2003). Evaluation of the dual route theory of reading: a metanalysis of 35 neuroimaging studies. Neuroimage 20, 693–712 10.1016/S1053-8119(03)00343-4 14568445

[B26] Josephs O. Henson R. N. (1999). Event-related functional magnetic resonance imaging: modelling, inference and optimization. Philos. Trans. R. Soc. Lond. B Biol. Sci. 354, 1215–1228 10.1098/rstb.1999.0475 10466147 PMC1692638

[B27] Kim H. (2011). Neural activity that predicts subsequent memory and forgetting: a meta-analysis of 74 fMRI studies. Neuroimage 54, 2446–2461 10.1016/j.neuroimage.2010.09.045 20869446

[B28] Kirwan C. B. Wixted J. T. Squire L. R. (2008). Activity in the medial temporal lobe predicts memory strength, whereas activity in the prefrontal cortex predicts recollection. J. Neurosci. 28, 10541–10548 10.1523/JNEUROSCI.3456-08.2008 18923030 PMC2590932

[B29] Kriegeskorte N. Simmons W. K. Bellgowan P. S. F. Baker C. I. (2009). Circular analysis in systems neuroscience: the dangers of double dipping. Nat. Neurosci. 12, 535–540 10.1038/nn.2303 19396166 PMC2841687

[B30] Loftus G. R. Masson M. E. J. (1994). Using confidence intervals in within-subject designs. Psychon. Bull. Rev. 1, 476–490 10.3758/BF03210951 24203555

[B31] McNab F. Klingberg T. (2008). Prefrontal cortex and basal ganglia control access to working memory. Nat. Neurosci. 11, 103–107 10.1038/nn2024 18066057

[B31a] Nelson D. L. McEvoy C. L. Schreiber T. A. (2004). The University of South Florida free association, rhyme, and word fragment norms. Behav. Res. Methods Instrum. Comput. 36, 402–427 10.3758/BF03195588 15641430

[B32] Otten L. J. Rugg M. D. (2001). When more means less: neural activity related to unsuccessful memory encoding. Curr. Biol. 11, 1528–1530 10.1016/S0960-9822(01)00454-7 11591321

[B33] Paller K. A. Wagner A. D. (2002). Observing the transformation of experience into memory. Trends Cogn. Sci. 6, 93–102 10.1016/S1364-6613(00)01845-3 15866193

[B34] Park H. Rugg M. D. (2008). Neural correlates of successful encoding of semantically and phonologically mediated inter-item associations. Neuroimage 43, 165–172 10.1016/j.neuroimage.2008.06.044 18675362 PMC2575045

[B35] Park H. Rugg M. D. (2011). Neural correlates of encoding within- and across-domain inter-item associations. J. Cogn. Neurosci. 23, 2533–2543 10.1162/jocn.2011.21611 21254802 PMC3309035

[B36] Park H. Shannon V. Biggan J. Spann C. (2012). Neural activity supporting the formation of associative memory versus source memory. Brain Res. 1471, 81–92 10.1016/j.brainres.2012.07.012 22800807

[B37] Park H. Uncapher M. R. Rugg M. D. (2008). Effects of study task on the neural correlates of source encoding. Learn. Mem. 15, 417–425 10.1101/lm.878908 18511693 PMC2414252

[B38] Prince S. E. Daselaar S. M. Cabeza R. (2005). Neural correlates of relational memory: successful encoding and retrieval of semantic and perceptual associations. J. Neurosci. 25, 1203–1210 10.1523/JNEUROSCI.2540-04.2005 15689557 PMC6725951

[B39] Ranganath C. Yonelinas A. P. Cohen M. X. Dy C. J. Tom S. M. D'Esposito M. (2004). Dissociable correlates of recollection and familiarity within the medial temporal lobes. Neuropsychologia 42, 2–13 14615072 10.1016/j.neuropsychologia.2003.07.006

[B40] Rugg M. D. Johnson J. D. Park H. Uncapher M. R. (2008). Encoding-retrieval overlap in human episodic memory: a functional neuroimaging perspective. Prog. Brain Res. 169, 339–352 10.1016/S0079-6123(07)00021-0 18394485

[B41] Rugg M. D. Vilberg K. L. Mattson J. T. Yu S. S. Johnson J. D. Suzuki M. (2012). Item memory, context memory and the hippocampus: fMRI evidence. Neuropsychologia 50, 3070–3079 10.1016/j.neuropsychologia.2012.06.004 22732490 PMC3472091

[B42] Sadeh T. Shohamy D. Levy D. R. Reggev N. Maril A. (2011). Cooperation between the hippocampus and the striatum during episodic encoding. J. Cogn. Neurosci. 23, 1597–1608 10.1162/jocn.2010.21549 20666593

[B43] Seghier M. L. Fagan E. Price C. J. (2010). Functional subdivisions in the left angular gyrus where the semantic system meets and diverges from the default network. J. Neurosci. 30, 16809–16817 10.1523/JNEUROSCI.3377-10.2010 21159952 PMC3105816

[B44] Slotnick S. D. Dodson C. S. (2005). Support for a continuous (single-process) model of recognition memory and source memory. Mem. Cogn. 33, 151–170 10.3758/BF03195305 15915801

[B45] Sommer T. Rose M. Weiller C. Büchel C. (2005). Contributions of occipital, parietal and parahippocampal cortex to encoding of object-location associations. Neuropsychologia 43, 732–743 10.1016/j.neuropsychologia.2004.08.002 15721186

[B46] Song Z. Jeneson A. Squire L. R. (2011). Medial temporal lobe function and recognition memory: a novel approach to separating the contribution of recollection and familiarity. J. Neurosci. 31, 16026–16032 10.1523/JNEUROSCI.3012-11.2011 22049444 PMC3227550

[B47] Sperling R. Chua E. Cocchiarella A. Rand-Giovannetti E. Poldrack R. Schacter D. L. (2003). Putting names to faces: successful encoding of associative memories activates the anterior hippocampal formation. Neuroimage 20, 1400–1410 10.1016/S1053-8119(03)00391-4 14568509 PMC3230827

[B48] Staresina B. P. Davachi L. (2006). Differential encoding mechanisms for subsequent associative recognition and free recall. J. Neurosci. 26, 9162–9172 10.1523/JNEUROSCI.2877-06.2006 16957073 PMC6674493

[B49] Summerfield C. Greene M. Wager T. Egner T. Hirsch J. Mangels J. (2006). Neocortical connectivity during episodic memory formation. PLoS Biol. 4:e128 10.1371/journal.pbio.0040128 16605307 PMC1436028

[B50] Thompson-Schill S. L. D'Esposito M. Aguirre G. K. Farah M. J. (1997). Role of left inferior prefrontal cortex in retrieval of semantic knowledge: a reevaluation. Proc. Natl. Acad. Sci. U.S.A. 94, 14792–14797 9405692 10.1073/pnas.94.26.14792 PMC25116

[B51] Thompson-Schill S. L. D'Esposito M. Kan I. P. (1999). Effects of repetition and competition on activity in left prefrontal cortex during word generation. Neuron 23, 513–522 10433263 10.1016/s0896-6273(00)80804-1

[B52] Tulving E. (1983). Elements of Episodic Memory. New York, NY: Oxford University Press

[B53] Uncapher M. R. Hutchinson J. B. Wagner A. D. (2011). Dissociable effects of top-down and bottom-up attention during episodic encoding. J. Neurosci. 31, 12613–12628 10.1523/JNEUROSCI.0152-11.2011 21880922 PMC3172893

[B54] Uncapher M. R. Otten L. J. Rugg M. D. (2006). Episodic encoding is more than the sum of its parts: an fMRI investigation of multifeatural contextual encoding. Neuron 52, 547–556 10.1016/j.neuron.2006.08.011 17088219 PMC1687210

[B55] Uncapher M. R. Rugg M. D. (2009). Selecting for memory? The influence of selective attention on the mnemonic binding of contextual information. J. Neurosci. 29, 8270–8279 10.1523/JNEUROSCI.1043-09.2009 19553466 PMC2730727

[B56] Uncapher M. R. Wagner A. D. (2009). Posterior parietal cortex and episodic encoding: insights from fMRI subsequent memory effects and dual-attention theory. Neurobiol. Learn. Mem. 91, 139–154 10.1016/j.nlm.2008.10.011 19028591 PMC2814803

[B56a] Van Essen D. C. (2005). A Population-Average, Landmark- and Surface-based (PALS) atlas of human cerebral cortex. Neuroimage 28, 635–662 10.1016/j.neuroimage.2005.06.058 16172003

[B57] Wagner A. D. Schacter D. L. Rotte M. Koutstaal W. Maril A. Dale A. M. (1998). Building memories: remembering and forgetting of verbal experiences as predicted by brain activity. Science 281, 1188–1191 10.1126/science.281.5380.1188 9712582

